# Balancing the Dilution and Oddity Effects: Decisions Depend on Body Size

**DOI:** 10.1371/journal.pone.0014819

**Published:** 2011-07-05

**Authors:** Gwendolen M. Rodgers, Jonathan R. Ward, Beth Askwith, Lesley J. Morrell

**Affiliations:** 1 Institute of Integrative and Comparative Biology, University of Leeds, Leeds, United Kingdom; 2 Department of Biological Sciences, University of Hull, Hull, United Kingdom; University of California San Diego, United States of America

## Abstract

**Background:**

Grouping behaviour, common across the animal kingdom, is known to reduce an individual's risk of predation; particularly through dilution of individual risk and predator confusion (predator inability to single out an individual for attack). Theory predicts greater risk of predation to individuals more conspicuous to predators by difference in appearance from the group (the ‘oddity’ effect). Thus, animals should choose group mates close in appearance to themselves (eg. similar size), whilst also choosing a large group.

**Methodology and Principal Findings:**

We used the Trinidadian guppy *(Poecilia reticulata),* a well known model species of group-living freshwater fish, in a series of binary choice trials investigating the outcome of conflict between preferences for large and phenotypically matched groups along a predation risk gradient. We found body-size dependent differences in the resultant social decisions. Large fish preferred shoaling with size-matched individuals, while small fish demonstrated no preference. There was a trend towards reduced preferences for the matched shoal under increased predation risk. Small fish were more active than large fish, moving between shoals more frequently. Activity levels increased as predation risk decreased. We found no effect of unmatched shoal size on preferences or activity.

**Conclusions and Significance:**

Our results suggest that predation risk and individual body size act together to influence shoaling decisions. Oddity was more important for large than small fish, reducing in importance at higher predation risks. Dilution was potentially of limited importance at these shoal sizes. Activity levels may relate to how much sampling of each shoal was needed by the test fish during decision making. Predation pressure may select for better decision makers to survive to larger size, or that older, larger fish have learned to make shoaling decisions more efficiently, and this, combined with their size relative to shoal-mates, and attractiveness as prey items influences shoaling decisions.

## Introduction

Group living is widespread across the animal kingdom, particularly in prey species, as it carries a number of proposed anti-predator benefits. These include the dilution of individual risk [1], [2], the many-eyes theory of increased vigilance [3], [4], and the confusion effect, where a predator has difficulty in targeting a specific individual for attack [5], all of which are increased in larger groups. The confusion effect is also enhanced when prey are morphologically and behaviourally similar [6], but when phenotypically distinct individuals occur within a group, predators preferentially target these individuals, enhancing their success [7], [8]. This is known as the oddity effect, and is thought to select for behaviours in prey that lead to the formation of phenotypically assorted groups. Assortment by species [9], body size [10], colour [11], [12], and parasite load [13] are all found. Preferences for groups containing kin [14] or familiar individuals [15] may also act as a mechanism to reduce oddity: kin may share inherited elements of their phenotype, while familiar individuals may have experienced the same recent environment or diet, which can affect phenotype [12], [16], [17].

Multiple factors contribute to an individual's decision to join one group over another, but we know little about how these factors interact to contribute to the complex decisions made by grouping animals. In natural circumstances, the characteristics of groups, such as size and composition, fluctuate alongside changing ecological variables. Some work has investigated the trade-offs between two attractive characteristics of groups: Swordtails (*Xiphophorus* spp) prefer shoals containing individuals of similar body size over those of dissimilar body size when each contains the same number of fish, but exhibit no preference between small size-matched shoals and large, unmatched shoals [18], while for mollies (*Poecilia latipinna*) body colouration is more important than shoal size [19]. Preferences for familiar fish are traded off against preferences for larger group size [20] and affected by recent diet [17], [21].

Ecological variables also play a key role in grouping decisions: the strength of preferences for particular groups is affected by distance to the group [22], food availability [23] and predation risk [23], [24]. Predation risk is a key ecological factor driving the evolution of morphological, behavioural and life-history traits. Under the threat of predation, aggregation tendency increases [2], [25], [26], [27] while groups may also become phenotypically homogenous [8], [24], [28], [29], suggesting increased importance of both dilution and oddity effects at increased predation risk. Increased predation risk also acts to reduce overall activity levels, firstly because prey movement may serve as a cue to predators and secondly because activities such as moving between groups means a period of isolation between shoals when risk is increased [30], [31]. This may impact on an individual's ability to sample the available groups and make the optimal social decision (giving the largest reduction in individual risk).

Here, we investigate how fish (Trinidadian guppies *Poecilia reticulata*) trade off the benefits of shoaling with a phenotypically similar shoal (reducing the oddity effect) against the benefits of shoaling with a numerically large group (increasing the dilution effect). As the benefits of both dilution and costs of oddity are likely to increase with increasing predation risk, we carry out our experiments on 7 populations that differ in the predation risk they experience, to investigate the effect of risk on the balance between dilution and oddity. We investigate preferences when individuals are offered a choice between a shoal of similar body size to themselves (the ‘matched shoal’) and one consisting of individuals of a different body size (the ‘unmatched shoal’), predicting that fish should preferentially associate with those of similar body size, and that the strength of this preference should increase as predation risk increases. We then increase the number of fish in the unmatched shoal and predict that either: 1) Under increased predation risk, dilution becomes increasingly important, and preferences for the size-matched shoal decrease in favour of the numerically larger shoal, or 2) under increased predation risk, the oddity effect becomes increasingly important, and preferences for the numerically larger shoal decrease in favour of the size-matched shoal. In addition, we investigated activity levels (moving between shoals) as this may impact on the ability to make shoaling decisions.

## Results

### Proportion of shoaling time spent with matched shoal

**Figure 1:**
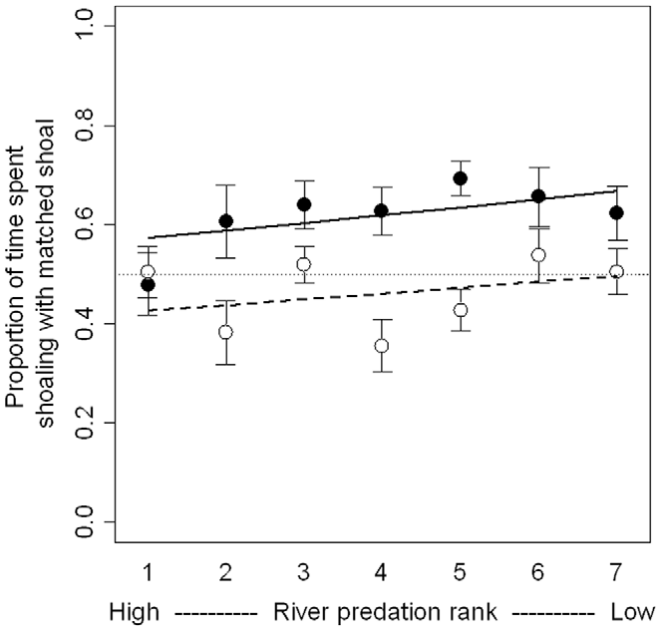
The proportion of time spent shoaling with the body size matched shoal. Solid lines and filled circles represent large test fish, dashed lines and open circles are small test fish. The dotted line at 0.5 represents no preference. Error bars represent ± 1 S.E. There is a significant effect of body size (p<0.001), as large fish show a stronger preference than small fish, and a marginal effect of predation risk (p = 0.059).

The proportion of shoaling time the focal fish spends with the body-size matched shoal, as opposed to the non-matched shoal (of increasing shoal size) is a measure of its preference for shoaling with fish of a similar body size. We found that larger-bodied test fish (large fish) showed a significantly stronger preference for the size-matched shoal compared to smaller-bodied test fish (small fish) (GLM, p<0.001, table 1, figure 1), but found only a marginal effect of predation risk (GLM, p = 0.059), no effect of the non-matched shoal size (GLM, p = 0.487, fig 1, table 1), and no significant interactions.

**Table 1:** Results of the generalized linear modelling analyses of the effects of predation risk, body size and unmatched shoal size on both response variables.

Response variable	Estimate	Std. Error	t	p
Proportion of time shoaling with size matched shoal				
Body size	−0.646	0.118	−5.492	<**0.001**
Predation risk	−0.056	0.030	1.897	0.059
Unmatched shoal size	−0.025	0.036	−0.697	0.487
Number of times zone lines were crossed				
Body size	0.290	0.071	4.075	<**0.001**
Predation risk	0.051	0.176	2.923	**0.004**
Unmatched shoal size	0.029	0.022	1.351	0.177

Significant p-values are presented in bold font.

We next tested whether the proportion of time spent with the size-matched shoal differed significantly from random, for all predation risk and shoal size treatments together. Fish choosing randomly between shoals are expected to spend, on average, 50% of their time with each shoal (illustrated by the horizontal dashed line in figure 1), with variance decreasing with increasing sample sizes.” Large fish showed a significant preference for the body-size matched shoal (one sample t-test, all shoal size treatments together, t = 3.44, p = 0.001, n = 63), whereas small fish showed no preference (one sample t-test, t = −0.65, p = 0.517, n = 66).

### Activity levels

We found that small fish were significantly more likely to cross the zone lines (switch shoals) than large fish (GLM: p<0.001, figure 1) and that shoal switching decreased with increasing predation risk (GLM: p = 0.004, figure 1) but no effect of unmatched shoal size treatment (GLM: p = 0.177) and no effect of significant interactions between these variables on this response variable (table 1).

## Discussion

Our prediction that fish should trade off the relative costs and benefits of associating with particular shoals, depending on predation risk, is partially supported by our results. Critically, however, we found that shoaling decisions are dependent upon individual body size, suggesting that the anti-predator benefits to grouping, such as a reduction in individual risk through dilution [2], oddity [7], [8] and confusion [7], [32] effects are dependent upon individual characteristics. A preference for associating with size-matched fish can be explained through the oddity effect [8], and has been found in previous studies [10], [33], [34]. Here, we found that only large-bodied fish showed a significant preference for the size matched shoal, suggesting an increased importance of oddity for large fish compared to small ones. Large guppies are likely to be preferentially targeted by their predators [25], as they have a higher calorific value than small guppies, and major predators such as *C. frenata* are unlikely to be gape limited [35]. This, combined with their increased conspicuousness to visual predators, may make phenotypic matching highly important for large fish. Although small fish may be more conspicuous in groups of larger ones, their lower value to predators may make them less likely to be targeted. An alternative explanation is that small female fish may be able to avoid sexual harassment by associating with larger females who may be more attractive to males [29], although we found no evidence that small fish preferred to shoal with larger ones, instead finding no preference. Further work is needed to distinguish between these two classes of explanation.

Our finding that body size influenced preferences for the size matched shoal may also be explained by examining activity levels. Small fish were much more likely to switch between the two stimulus shoals than large fish, and this switching was reduced at high predation levels. In addition to being a measure of activity [36], moving between shoals may also represent the ‘dynamic shoaling tendency’ of an individual, and be an indicator of how the fish samples the available shoals (or ‘changes its mind’). Multiple sampling of shoals by small fish may lead to more equal distribution of time spent with each shoal, and may be explained by two hypotheses: 1) a *learning hypothesis* and 2) a *predation hypothesis*.

**Figure 2:**
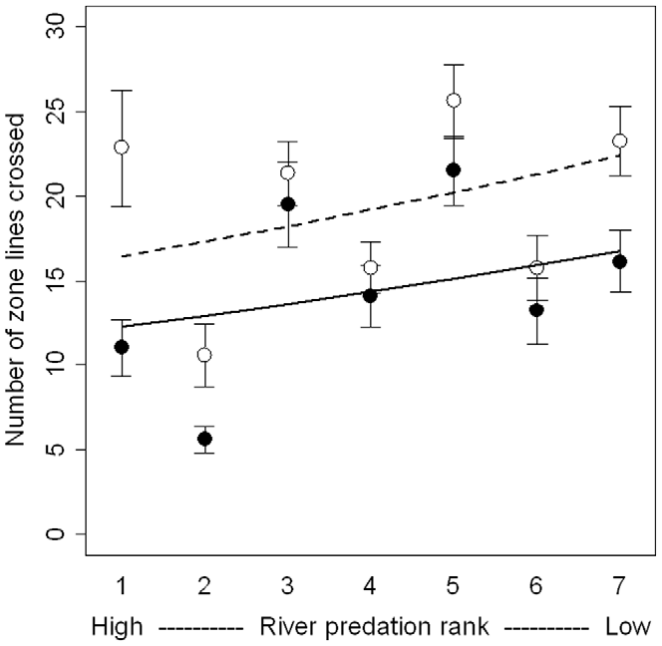
The number of times the test fish crossed the zone lines (switched shoals). Solid lines and filled circles represent large test fish, dashed lines and open circles are small test fish. Error bars represent ± 1S.E. There is a significant effect of predation risk (p = 0.004) and body size (p<0.001), but no interaction. Small fish swap shoal more often than large fish overall.

The learning hypothesis suggests that larger (and therefore older) fish have more experience in making shoaling decisions and therefore need to sample each shoal fewer times than small fish before making a decision. Evidence suggests that fish do learn to make shoal choices as they grow older, as juvenile guppies less than 18mm in length (50 days old) cannot distinguish between large and small shoals [37]. The predation hypothesis derives from the idea that moving between shoals increases predation risk [30], [31], and suggests that rapid decision making acts as an anti-predator tactic. Slower decision-makers may have fallen victim to predators meaning that large fish in natural populations are the ones that are able to make decisions rapidly. This is supported by our finding that activity levels are lower in the highest risk populations (figure 2), where shoaling preferences are closer to random (figure 1). This suggests an evolutionary pressure towards rapid decision making associated with predator avoidance, which may influence the way animals choose between shoals of differing characteristics and balance conflicting preferences for dilution versus oddity effects. Both the learning and predation risk hypotheses may provide alternative explanations as to why smaller fish showed weaker preferences for the size matched shoal than large fish.

We found a marginal trend towards a decreased preference for the matched shoal over the unmatched one at higher predation risks for both small and large fish, suggesting that as risk increases, the oddity effect decreases in importance, and instead, simply being in any group is important. In the wild, oddity may arise in various ways as individuals differ in both appearance and behaviour. Variation in these traits within a group may also affect the strength of any oddity effects: if groups are variable in particular characteristics, oddity may be of reduced importance, or oddity in one trait may be of lower importance than in another.

In contrast to some previous studies we found no effect of the size of the unmatched shoal on preferences. Sticklebacks (*Gasterosteus aculeatus*) switch preferences to a larger shoal over a smaller, body-size matched shoal [38] as the benefits of dilution begin to outweigh the benefits of reduced oddity, and swordtails (*Xiphophorus* spp) show preferences for both size-matched shoals and larger shoals, but when faced with a conflict between these preferences, show no preference for either shoal [18]. Fish in our study showed no change in preference with increasing shoal size, suggesting that the increased dilution effect in larger shoals was of limited importance at the shoal sizes we tested. Incorporating larger shoal sizes for the unmatched shoal may have provided greater dilution-related benefits to associating with an unmatched shoal.

### Conclusion

Our results indicate that social decisions in the guppy are dependent upon the body size of the individual. Predation risk and body size of individuals within a shoal act together to influence shoaling decisions. The resolution of trade-offs in behaviour is often complex [39], [40], and while our results demonstrate that shoaling priorities are influenced by an interaction between predation risk, shoal characteristics and body size, other factors may also influence these decisions. Familiarity [41], competitive ability [42], dominance status [43], parasite load [44] and whether foraging is a priority [23] are all known to affect social decisions, and may interact with the factors studied here. Sex may also play a role: larger shoal sizes are more important for female zebrafish (*Danio rerio*) than for males [45], and males may join a group for both anti-predator and reproductive reasons [15], complicating decisions further. The effectiveness of these anti-predator tactics in reducing predation may also depend on predator characteristics, in particular hunger, but also sensory capabilities and physical condition. There is the potential for fascinating future work understanding these complex social and anti-predator decisions.

## Methods

We carried out this study at the University of the West Indies Biology Department laboratories in Tunapuna, Trinidad, using guppies from seven well known field sites in the rivers of the northern mountain range (table 2), representing a range of predation risks for the guppy [35], [46]. Guppies are sympatric with a variety of aquatic predators: the most significant of these is *Crenicichla frenata* which is large enough to consume even the largest guppies. Other, more minor guppy predators include *Aequidens pulche*r, *Rivulus hartii* and *Macrobrachium* spp (a freshwater prawn) [35], [46], [47]. However, the presence of waterfalls prevents the upstream migration of the major guppy predators, providing a range of predation environments [35]. Previous comparative work on different guppy populations has demonstrated between-population differences in morphology, behaviour and life-history, and this has been largely attributed to differences in the predation risk experienced by these populations [47]. Predation risk was assessed in each of the seven different populations, following the methodology described in [35] and [46], and outlined below. This method provides a simple way of assessing predation risk, which remains broadly consistent between years [46]). It is also consistent with the alternative method of classifying populations as ‘high’ or ‘low’ predation based on the presence or absence respectively of *Crenicichla frenata* [48].

**Table 2:** Study sites (rivers), their geographical location, the predator species observed approaching the confined stimulus female, the mean number of predator approaches to the container (mean abundance) of the predator across 5 pools, and the predation risk rank assigned to the river with 1 as the highest risk.

River	Grid Reference		Predators present	Mean abundance	Predation risk rank
	North	West			
Lower Aripo	10^o^40′	61^o^14′	*Crenicichla frenata Aequidens pulcher Astyanax bimaculatus*	77.2 1.6 71.0	1
Tacarigua	10^o^41′	61^o^22′	*Crenicichla frenata Aequidens pulcher*	48.0 8.2	2
Lower Turure	10^o^40′	61^o^10′	*Crenicichla frenata Aequidens pulcher Astyanax bimaculatus*	12.6 3.0 26.0	3
Arima	10^o^41′	61^o^17′	*Crenicichla frenata*	11.0	4
Arouca	10^o^40′	61^o^19′	*Crenicichla frenata Astyanax bimaculatus*	2.2 0.2	5
Upper Turure	10^o^41′	61^o^10′	*Rivulus hartii Macrobracium* spp.	23.8 0.2	6
Upper Aripo	10^o^41′	61^o^14′	*Rivulus hartii*	2.8	7

### Assessment of predation risk

Stimulus guppies were caught from a downstream site in each river using a hand seine net. A female stimulus guppy (25–30 mm) was restrained in a clear container (diameter: 80 mm, height: 110 mm) pierced with approx 50 holes (2 mm diameter) providing both visual and olfactory cues to potential predators. The container was weighted with gravel to hold it in position on the river bed was attached to a monofilament line attached to allow for positioning and removing the bottle while minimizing disturbance. The container was placed in a pool (over 30 cm deep) in the river, and after a 10 minute acclimatization period, we recorded approaches by all non-guppy fish. An approach was defined as an individual moving to within three body lengths of the bottle, with the head orientated towards it. Observations were made every 10 seconds for a 10 minute period. Fish species and number were recorded. The observer was positioned on the river bank over 2 m from the container prior to the acclimatization period, with a recorder positioned further away. Observations took place in daylight, between the hours of 12:00 and 17:00. To estimate risk over the whole river, five pools in each river were observed on the same day, each one at least 5 m upstream from the previous, and a different stimulus female was used in each pool. The ‘abundance’ measure here is certainly more than the number of separate individual predators observed, and repeats of individuals at different time points are recorded as separate counts. We assume that a predator which remains near the stimulus guppy, or returns to it represents a greater threat, and this is reflected in the risk ranking.

Predation risk was assessed according to the abundance of the various predators observed, in order of risk to guppies [35], [46], [49]: *C. frenata* is considered the most dangerous, followed by *A. pulcher*, *R. hartii* and finally *Macrobrachium* spp. Predator abundances and ranks assigned to each river are shown in table 2. Rivers were first ranked according to abundance of the most dangerous predator, *C. frenata* (the mean number of approaches to the stimulus guppy recorded across the 5 pools for each river; ‘mean abundance’ in table 2). The river with the highest mean abundance (the Lower Aripo) was ranked as 1, and so on until the river with the lowest abundance of *C. frenata*, the Arouca, ranked 5. In the remaining 2 rivers, *C. frenata* was not observed and so these were given rankings according to the abundance of *R. hartii*, as none of the rivers observed contained *A. pulcher* in the absence of *C. frenata*. Other fish species approaching the container but thought to pose no or a very low risk to guppies [47] were *Astyanax bimaculatus*, *Hemibrycon* spp. and *Hypostomus robinii*. Although this method uses a ranking system to assess risk rather than providing a specific measure of risk, it allows a graded assessment of predation risk which provides greater insight than classification as ‘high’ or ‘low’, as it takes into account possible variation in predator numbers within a class [35], [46].

### Fish Capture and husbandry

At each of the seven field sites, we collected approximately 60 small (17–22 mm) and 60 large (27–32 mm) female guppies from a 100 m continuous stretch of river (containing no barriers to guppy dispersal, such as waterfalls). We collected fish from the same stretch of river that had been used to assess predation risk, but on a subsequent day. Fish were caught with small seine and hand nets, and transported back to the laboratory where they were placed into large holding aquaria (approx. length x width x height 90×35×50 cm) and allowed to settle overnight to reduce stress. The day following collection, we sorted the fish into ‘large’ and ‘small’ size classes. Fish were measured (±1 mm) using electronic callipers. We used only female guppies in these experiments, as they form the core of naturally occurring shoals [15]. Male guppies display less shoal fidelity [50] and using only female fish controls for any confounding effect of sexual behaviour.

Once sorted, fish were held in glass tanks (60×30×30 cm) divided into two with green square mesh (hole size 1 mm). One side of each tank contained large fish, and the other contained small fish from a single population. The mesh screen ensured that the fish were physically separated, but remained in visual and olfactory contact with each other. Each population was divided into two separate tanks, one containing test fish and the other containing stimulus fish. In total we used four holding tanks, allowing fish from two populations to be held and tested at once. Further populations were collected and tested once experiments on each pair of populations were completed. Tanks were covered on three sides with black opaque material to ensure no visual contact between fish in separate tanks, and to reduce stress. Fish were housed in three day aged aerated water to a depth of 15 cm, and fed *ad libitum* on dry flake food at the end each day to avoid the effects of satiety on response [51]. Trials were conducted on the three days following size sorting. Laboratory conditions were maintained at a 12 h light: 12 h dark cycle at 25°C to replicate conditions in the wild.

### Binary Choice Trials

Trials were conducted in glass aquaria (60×30×30 cm) filled to a depth of 10 cm with aerated three day aged water, covered with black opaque material on all four sides. We used a standard binary choice design [51], where two shoals of stimulus fish were contained in transparent plastic cylinders (7 cm diameter) placed in the test aquarium. These cylinders were perforated to allow chemical cues from the stimulus shoals to pass through, and positioned at opposite ends of the choice tank, so that their centres were 15 cm from the tank end and two sides. Each contained white gravel to a depth of 1 cm to ensure that they did not move during the trial. Circular preference zones were marked on the underside of the tank 4 cm and 6 cm from the edge of the cylinder (equivalent to approx. 2 standard body lengths for small and large fish respectively) which results in a conservative estimate of shoaling tendency [52].

Both large and small fish test fish were presented with two stimulus shoals, one consisting of large fish, and one of small fish. We defined the shoal of fish of similar body size to the test fish as the ‘matched shoal’ and the shoal that differed in body size to the test fish as the ‘unmatched shoal’. Thus for small fish, the matched shoal consisted of other small fish, and the unmatched shoal consisted of large fish, and vice versa for large fish. The matched shoal always contained 4 fish, and we investigated three different shoal sizes for the unmatched shoal: 4, 6 or 8 fish, giving a total of 6 different treatments overall (three shoal sizes for two body size classes of the focal fish). These are summarised in table 3. Twenty focal fish of each size class from each river were tested in each shoal size treatment.

**Table 3:** Summary of experimental treatments.

Matched stimulus shoal	Test fish	Unmatched stimulus shoal
4×Large fish	1×Large fish	4×Small fish
4×Large fish	1×Large fish	6×Small fish
4×Large fish	1×Large fish	8×Small fish
4×Small fish	1×Small fish	4×Large fish
4×Small fish	1×Small fish	6×Large fish
4×Small fish	1×Small fish	8×Large fish

In each trial, stimulus shoals were placed into the cylinders with hand nets and the test fish was introduced to the centre of the tank, equidistant from both stimulus shoal cylinders. The fish were given 15 minutes to acclimatize before the trial began. Trials lasted 10 minutes, and were observed by a stationary observer from above, to ensure that the point at which zone lines were crossed could be accurately observed, and to reduce disturbance to the fish. Cumulative time in each preference zone was measured using stopwatches. We also recorded the number of times the test fish moved between preference zones, as a measure of their activity levels or dynamic shoaling tendency. Half of the water in the binary choice tank was changed after each trial, to reduce the build up of olfactory cues. After the trial, fish were returned to holding tanks. Test fish were used in only one trial, and were then added to the pool of stimulus fish. Stimulus fish were chosen randomly from this pool for each trial.

### Statistical Analysis

Data were analyzed using the statistical analysis program ‘R’ (v. 2.6.0; R Core Development Team 2007). We analyzed two response variables: the proportion of shoaling time spent with the body size-matched shoal and the number of times zone lines were crossed. For each response variable, we investigated the effects of body size, unmatched shoal size and predation risk, and their two- and three-way interactions on the behaviour of the test fish using generalized linear modelling (GLM). In each case, we used suitable error distributions (quasi distributions were necessary for each to account for over-dispersion; Poisson errors for number of zones crossed, and binomial errors for the proportion of shoaling time spent with the matched shoal). Non-significant interactions were dropped from the analysis to produce the minimum adequate model.
